# Curiosities of the Microscope, or Illustrations of the Minute Parts of Creation, Adapted to the Capacity of the Young, with Colored Illustrations

**Published:** 1852-11

**Authors:** 


*Curiosities of the Microscope, or illustrations of the minute parts of creation, adapted to the capacity of the young, with colored illustrations.* By Rev. J. A. Wythes, M. D. Philadelphia: Lindsay & Blakiston.

This is a neat little volume, very prettily gotten up, and containing much to interest those for whom it is intended. It scarcely comes within the range of a medical journal, however, though it presents much to attract those to whom it is addressed.

